# A VLSI Chip for the Abnormal Heart Beat Detection Using Convolutional Neural Network

**DOI:** 10.3390/s22030796

**Published:** 2022-01-21

**Authors:** Yuan-Ho Chen, Szi-Wen Chen, Pei-Jung Chang, Hsin-Tung Hua, Shinn-Yn Lin, Rou-Shayn Chen

**Affiliations:** 1 Department of Electronics Engineering, Chang Gung University, Taoyuan 333, Taiwan; chenyh@mail.cgu.edu.tw (Y.-H.C.); nimbus0608@gmail.com (P.-J.C.); b0925123511@gmail.com (H.-T.H.); 2 Department of Radiation Oncology, Chang Gung Memorial Hospital-LinKou, Taoyuan 333, Taiwan; 3 Neuroscience Research Center, Chang Gung Memorial Hospital-LinKou, Taoyuan 333, Taiwan; cerebrum@ms13.hinet.net; 4 Department of Radiation Oncology, Chang Gung Memorial Hospital, Chang Gung University, Taoyuan 333, Taiwan; rt3126@gmail.com; 5 Department of Medical Imaging and Radiological Sciences, College of Medicine, Chang Gung University, Taoyuan 333, Taiwan

**Keywords:** very large scale integration implementation (VLSI), electrocardiogram (ECG), convolutional neural network (CNN)

## Abstract

The heart is one of the human body’s vital organs. An electrocardiogram (ECG) provides continuous tracings of the electrophysiological activity originated from heart, thus being widely used for a variety of diagnostic purposes. This study aims to design and realize an artificial intelligence (AI)-based abnormal heart beat detection with applications for early detection and timely treatment for heart diseases. A convolutional neural network (CNN) was employed to achieve a fast and accurate identification. In order to meet the requirements of the modularity and scalability of the circuit, modular and efficient processing element (PE) units and activation function modules were designed. The proposed CNN was implemented using a TSMC 0.18 μm CMOS technology and had an operating frequency of 60 MHz with chip area of 1.42 mm${}^{2}$ and maximum power dissipation of 4.4 mW. Furthermore, six types of ECG signals drawn from the MIT-BIH arrhythmia database were used for performance evaluation. Results produced by the proposed hardware showed that the discrimination rate was 96.3% with high efficiency in calculation, suggesting that it may be suitable for wearable devices in healthcare.

## 1. Introduction

Heart disease has become the leading cause in the top 10 causes of death globally. In order to effectively reduce mortality from sudden cardiac death, an early warning system for cardiac disease may be necessary. With regard to the noninvasive diagnostically useful tools for identifying patients at high risk of sudden cardiac death, artificial-intelligence-based medical electronic equipment technology, including wearable devices, has been overwhelmingly and continuously explored and developed in recent years. Over many aspects of such applications, electrocardiography (ECG) examination has been considered as a mature and well-established monitoring method since it provides electrophysiological signals emitted from the heart muscle. In fact, a variety of abnormal heart beats, for example, may be identified simply by analyzing the ECG signals.

There are a number of previous studies in the literature showing that ECG can be utilized for the development of noninvasive algorithms for accurately detecting various types of abnormal or premature heart beats, including lethal arrhythmias such as ventricular tachycardia and ventricular fibrillation [1,2,3,4,5]. In addition, some researchers have dedicated their efforts to the circuit-design-based studies aiming at the detection of abnormal ECG signals using the system-on-chip (SoC) platforms [6,7,8,9].

A machine-learning-assisted cardiac sensor SoC (CS-SoC) was developed for mobile healthcare applications [6]. According the results reported in this work, the CS-SoC achieved 95.8% in accuracy for ECG-based arrhythmia detection in a real-time manner with the only 48.6μW in power consumption. In [7], a low-power biological signal acquisition and classification system for human sensor networks is introduced; the SoC mainly consists of three modules: a high-pass sigma delta modulator-based biosignal processor, a switch key control transceiver, and a digital signal processor. In addition, another example of a wearable cardiac arrhythmia early detection system as proposed by [8] is implemented in FPGA. Numerical detection results produced by the MIT-BIH ECG arrhythmia database show that the proposed system may achieve a sensitivity and specificity of 94.64% and 99.41%, respectively. Moreover, a convolution neural network (CNN)-based premature ventricular complex (PVC) detection chip is proposed by [9]. Comprising two convolution layers and one fully connected layer, the CNN allows a fast training, and thus, the identification model can be then effectively established, achieving a detection accuracy of 94.94%.

Due to an increasing interest of medical wearable monitoring equipment nowadays, this research aims at the development of a CNN-based real-time ECG signal detection circuit to achieve an efficient and accurate abnormal heartbeat classification/detection. In general, the proposed circuit utilizes three convolution layers and two fully connected layers to classify six different types of ECG heartbeats, including the normal one. It should be noted that here the MIT-BIH arrhythmia database is adopted for network training and testing. The proposed CNN chip was implemented in a TSMC 0.18 μm complementary metal-oxide-semiconductor (CMOS) process to achieve a small area and high-speed design that may be well compatible with wearable ECG electrodes [10,11] and is thus very suitable for portable and wearable devices. In addition, it should be also noted that motion artifact is a big challenge for wearable ECG monitoring. In this aspect, some previous studies in the literature have indicated that motion artifact can be efficiently removed by deep learning models, including CNN [12,13]. Therefore, with some appropriate arrangements, we believe that the proposed CNN chip can be further applied to the task of ECG heart beat detection for wearable devices in healthcare. As a results, the application specific integrated circuit (ASIC) dissipates the power by 4.4 mW at the operating frequency of 60 MHz with a chip area of 1.4 mm${}^{2}$.

This paper is organized as follows: The overall structure of the CNN algorithm and the details in the architectural design of the proposed circuit are presented in Section 2. Section 3 provides the descriptions of a performance evaluation and comparison with a discussion. Conclusions are then briefly drawn in Section 4.

## 2. Proposed Method and Architecture

### 2.1. Proposed Method

In general, a typical ECG signal consists of five characteristic points, denoted as P, Q, R, S, and T, respectively. Different diseases manifest themselves by components formed by a variety of combinations of these characteristic points, such as the QRS complex, PR interval, ST segment, and so on. In addition, the variability of interbeat intervals (i.e., RR intervals), alternatively known as the heart rate variability, can be diagnostically useful for serving as a predictor of mortality after myocardial infarction. When an abnormality is detected at early stage, patients can be treated on a timely basis. The database used in this research was drawn from the MIT-BIH Arrhythmia Database [14]. It contains 48 two-channel ambulatory ECG recordings obtained from 47 subjects studied by the BIH Arrhythmia Laboratory between 1975 and 1979, and each recording is 30-min long. The recordings were sampled by 360 Hz with 11-bit resolution over a 10 mV range. For each ECG recording, the database also includes the computer-readable reference annotations for each beat, thus being very suitable for the applications into the task of ECG classification.

Our study aims to design and realize a CNN-based abnormal heartbeat detection ASIC, and the chip is capable of identifying six different types of ECG signals drawn from the MIT-BIH arrhythmia database as: normal, left bundle branch block (LBBB), right bundle branch block (RBBB), premature ventricular contraction (PVC), atrial premature beat (APB), and paced beat, as tabulated in Table 1.

**Figure 1:**
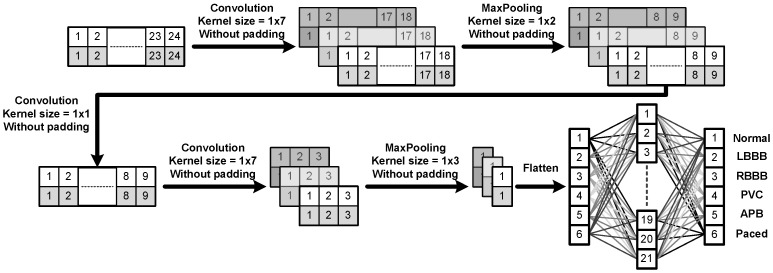
The schematic block diagram of the entire flow of the proposed CNN algorithm for ECG heart beat classification.

Figure 1 provides a schematic block diagram illustrating the entire flow of the proposed CNN algorithm for ECG heart beat classification. In order to reduce the circuit area, here we only included two convolutional layers to perform the main calculations and inserted an extra convolutional layer between both layers in the design of the CNN architecture, as shown in Figure 1. Since the kernel size of the extra convolutional layer is 1×1, this design would result in a significant reduction in the number of parameters, meaning the implementation of the circuit becomes much simpler while the overall training accuracy would still maintain at an adequately high level. To have a better idea about how the proposed CNN algorithm works, we here just take the third convolution layer as an example to illustrate how the convolution calculation performs and explain why it would produce three 2×3 blocks after the convolution. First, it should be noted that there are three 1×7 kernel filters at this layer. In addition, note that the input is a 2D array of size 2×9. The convolution is performed as follows. We slide each 1×7 kernel filter column-wise first and then advance along the rows over the input. Since each row of the input consists of only 9 data points and the filter length is 7, without zero padding, it would then result in a 2D output of size 2×3 after the filtering process. As a result, because there are three 1×7 kernel filters, it would finally produce three 2×3 blocks as outputs after the convolution calculation at this layer is completed. Moreover, in order to achieve the reusability in circuit design, we further analyzed all the calculations required by the convolutional layers as well as the fully connected (FC) layer and then determined the total number of the filter coefficients for the entire network structure. As a result, the total number of filter coefficients or parameters required for each layer of the proposed CNN structure are as listed in Table 2. Note that here in Table 2 “3×1×7” in the first convolution layer means that there are three kernel filters of size 1×7 in this layer.

### 2.2. Proposed Architecture

As described in previous subsection, when an ECG signal is input into the neural network, two-dimensional convolution calculations of three 1×7 filters in the first layer are performed and then followed by the 1×2 max pooling process. Note that a max pooling operation simply reduces the feature dimension by replacing the corresponding local patch in the convolutional layer of the same stage by its maximum value [15]. Next, a 1×1 filtering process in the second layer is then performed. Afterward, similar to the first layer, in the third layer, the convolution calculations of three 1×7 filters are performed and then followed by a 1×3 max pooling. Finally, the learned feature maps are flattened and then fed into a fully connected neural network with an input layer of 21 nodes and an output layer of 6 nodes (i.e., six types of different ECG heartbeats) obtained after the Softmax calculation. Figure 1 shows the schematic diagram of the proposed CNN algorithm. It should be noted that the main problem in implementing neural networks is that the detection accuracy, number of neurons, and the circuit area affect one another. For example, in order to enhance the detection accuracy, the number of neurons is unavoidably bound to increase, and thus, the original circuit area would undesirably increase.

**Figure 2:**
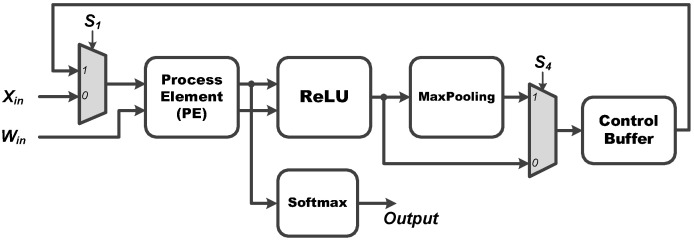
The overall hardware architecture of the main CNN core as proposed in this study.

Therefore, the proposed research aims to design and implement a very large scale integration (VLSI) circuit of the proposed CNN algorithm which can be used for ECG heartbeat classification. In order to substantially reduce the circuit area, here a modular design is adopted. Figure 2 shows the overall hardware architecture of the proposed main CNN core. It executes all the operations such as multiplication, addition, activation function, maximum pooling, and softmax calculations, as required by Figure 1. As depicted in Figure 2, the proposed CNN circuit mainly consists of process element (PE), ReLU, Softmax, MaxPooling, and Control Buffer modules. The PE module is essentially used for all multiplication-accumulation (MAC) operations. The activation function employs ReLU and Softmax modules. In addition, after each layer of CNN, the max pooling layer is generally applied. Finally, a temporary storage space is designed for storing the intermediate values produced by each layer. Consequently, under a constraint of small area, the modules as mentioned above are all designed to be repeatedly used so that the circuit may effectively and efficiently achieve a utilization maximization and the chip area may be thus substantially reduced. The details in these five modules are discussed as follows.

**Figure 3:**
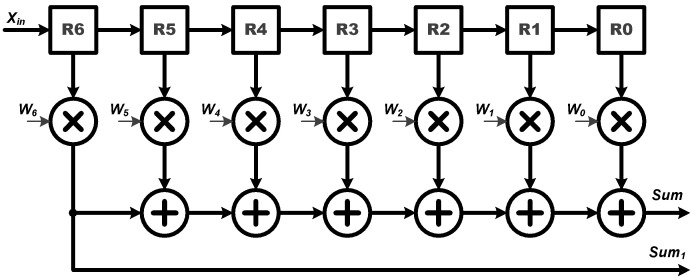
Architecture of the proposed PE module.

**Figure 4:**
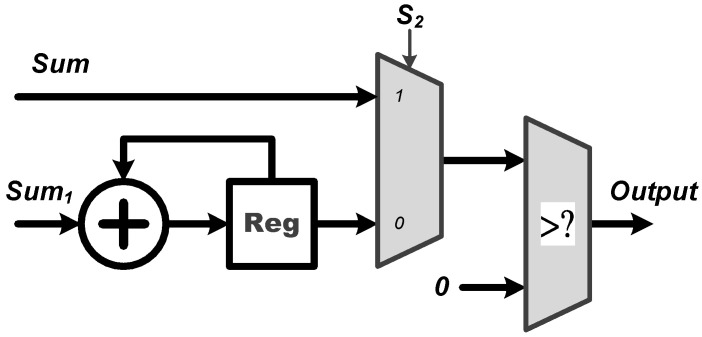
Architecture of the proposed ReLU module.

**Figure 5:**
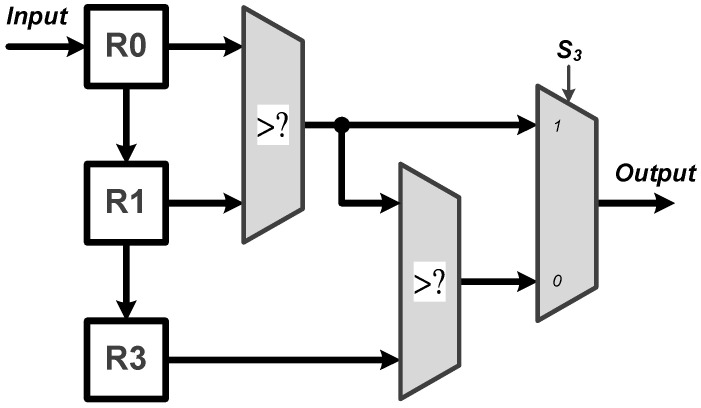
Architecture of the proposed MaxPooling module.

**Figure 6:**
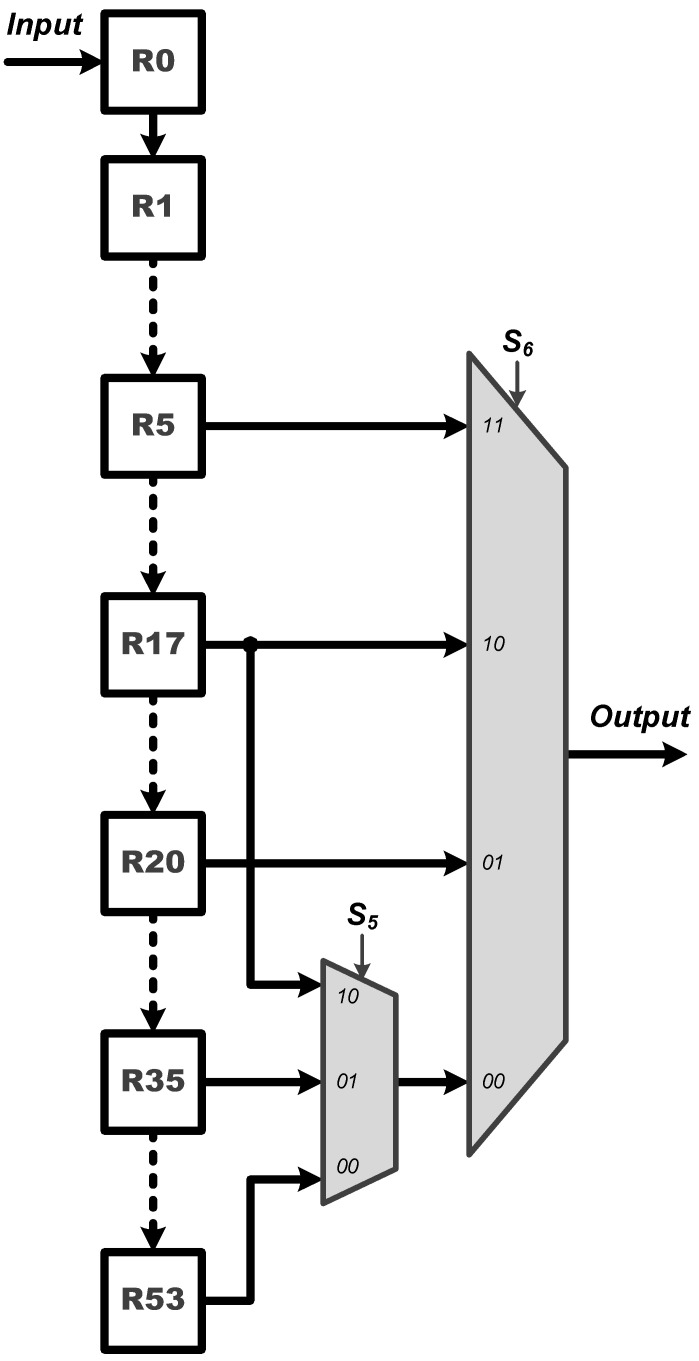
Architecture of the proposed Control Buffer module.

**Figure 7:**
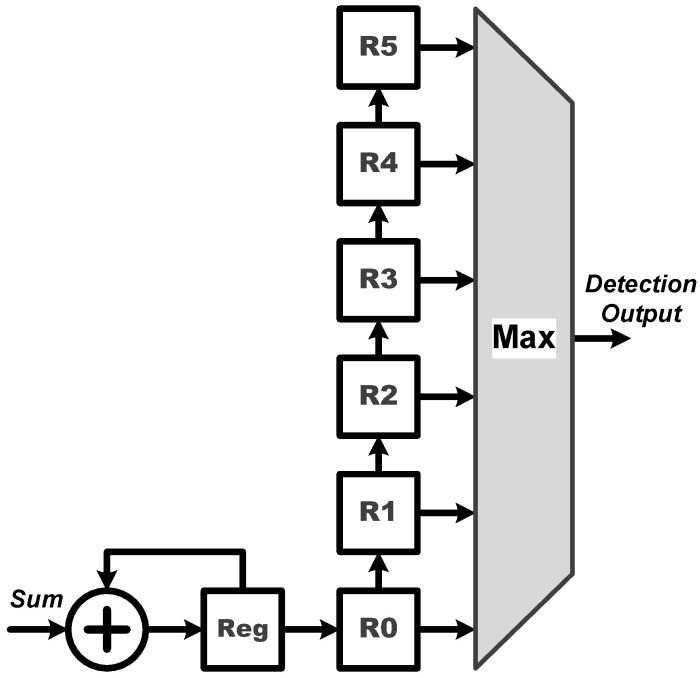
Architecture of the proposed Softmax module.

- **Process Element (PE)**: The PE module is employed for all the multiplication and addition (MAC) operations of the proposed CNN circuit. As shown in Figure 3, the proposed PE module executes the CNN filter size of 1×7 and 1×1, and the fully connected layer operation of 6 to 21 and 21 to 6. It should be noted that the proposed PE module includes seven multipliers and six adders and thus, a complete fully connected operation would include three executions of the PE module for finishing doing the 21 MAC operations. Moreover, **W**, where **W** = ${\left[W_{0},W_{1},W_{2},W_{3},W_{4},W_{5},W_{6}\right]}^{T}$, can be either a set of filter coefficients or a set of weights; **W** is a set of filter coefficients when the PE performs the convolution calculations while it is a set of weights when the PE performs the MAC calculations at the fully connected layer.
- **ReLU**: The activation function of ReLU outputs the positive number after the CNN operations. As shown in Figure 4, the proposed ReLU module uses the $S_{2}$ signal to select the results from 1×7 or 1×1 CNN operations and send them into the ReLU operation. We utilize a adder and register (Reg) to implement the operation of 1×1 CNN.
- **MaxPooling**: Max pooling includes two specifications of maximum pooling calculations 1×2 and 1×3, which are respectively after the first layer of CNN and the third layer of CNN. As shown in Figure 5, the proposed MaxPooling module shares the same hardware resource and uses the $S_{3}$ signal to select the result of 1×2 and 1×3 operations.
- **Control Buffer**: The proposed control buffer is the value temporary storage area which consists of 54 registers as shown in Figure 6. The $S_{5}$ signal is used to control data feeding into the PE to calculate 1×1 CNN operation, and the selection signal $S_{6}$ switches the data sending into PE to calculate 6×21 FC, 1×7 CNN, 21×6 FC, and 1×1 CNN operations.
- **Softmax**: The proposed softmax module calculates the maximum value as the detection output. An adder and a register (Reg) accumulate three results from the PE module to calculate the final layer of the 21×6 FC operations, as shown in Figure 7.

In addition, note that the calculation of the final FC layer is executed by the PE and softmax modules, as shown in Figure 2 and Figure 7, respectively. Since this final FC layer is in a neural network structure of size 21×6, there are 21 MAC operations required for producing the numerical result of each of the six output nodes before softmax operation. Considering our design in Figure 2, one may see that the proposed PE module has seven multipliers and six adders. Therefore, in order to produce the numerical result of each output node, the PE module should execute exactly three times, thus resulting in the required 21 MAC operations in total; each time when the result of one of the three PE executions is obtained, it is immediately sent to the softmax module, as indicated by “sum” in Figure 7. Further observing Figure 7, one may see that there are a register and an adder in the proposed softmax module. In fact, the register and adder are used to accumulate the results of the three PE executions to obtain the final numerical result of each output node at the final 21×6 FC layer. Once the weighted sums of all the six output nodes are obtained, the numerical results of all the six output nodes are stored in the registers R0, R1, R2, R3, R4, and R5, respectively. Next, the maximum value of all these weighted sums stored in the register array is then found so the final ECG heartbeat classification result as the detection output is finally determined.

**Figure 8:**
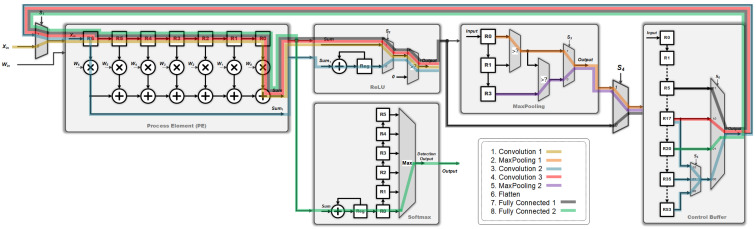
The operation for each layer in the proposed circuit.

In the proposed circuit, there are six multiplexers (MUXs) to control each layer’s operations as tabulated in Table 2. Table 3 shows the designated values of the select input(s) of each multiplexer when operating at different layers. Taking the convolution operation of the first layer as an example, we only need to set the values of $S_{1}$ and $S_{2}$ to 0 and 1, respectively, and the other multiplexers can be ignored. In this way, the PE module can be used to calculate 1×7 convolution operations of the first convolution layer. The rest of the calculations can be also completed in accordance with the settings in Table 3 using the proposed circuit. Obtaining this, the hardware resources of the proposed circuit can be shared. Figure 8 illustrates the data flow of each operation in the proposed circuit.

## 3. Results and Discussion

### 3.1. VLSI Chip Implementation

**Figure 9:**
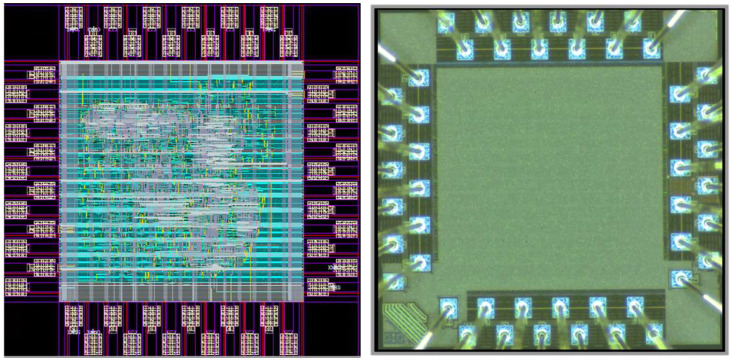
The layout (**left**) and photomicrograph (**right**) of the proposed CNN accelerator.

The chip of the proposed CNN accelerator was entrusted to Taiwan Semiconductor Research Institute (TSRI) to tape out using the 1.8-V TSMC 0.18-μm 1P6M CMOS process technology. The Synopsys Design Compiler was used to synthesize the RTL code, and the Cadence Innovus was then used for placement and routing. The proposed core was operated at a frequency of 60 MHz with power consumption of 4.4 mW. The chip area was 1.42 mm${}^{2}$. In addition, the layout and photomicrograph of the proposed core and its characteristics are as shown in Figure 9 and listed in Table 4, respectively.

**Figure 10:**
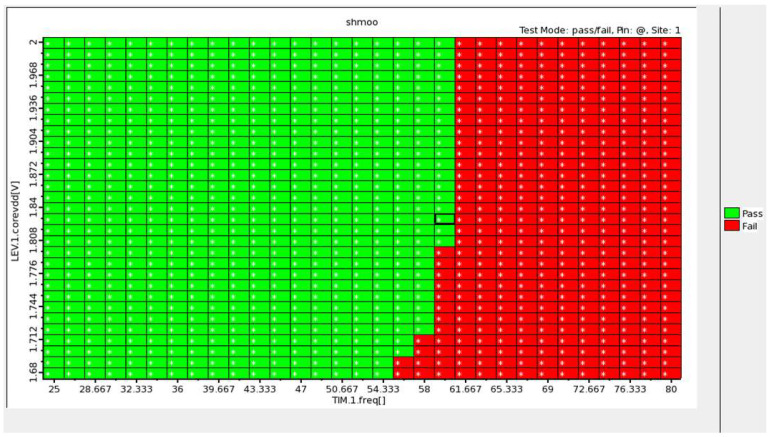
Shmoo plot of measurement results.

In addition, we also employed an Advantest V93000 equipment to verify the function of the proposed chip. The testing steps are described as follows: First, the ECG data drawn from the MIT-BIH Arrhythmia Database were loaded to the Advantest V93000 equipment. Next, the Advantest V93000 generated the ECG signals and input them into the proposed chip and then measured the output data produced from the chip. We also loaded the fixed-point software simulation results from MATLAB to the Advantest V93000 so that both the hardware and software simulation results can be compared. Figure 10 provides a shmoo plot of the measurement results for the chip. In the shmoo plot, a square in green color called “pass” indicates that both the hardware and software results were the same. According to the measurement results as shown in Figure 10, one may see that under a core voltage of 1.8 V, the maximal operating frequency that the chip can achieve is 60 MHz.

### 3.2. Comparison with the Existing Works

Table 5 provides a performance comparison among a number of existing works, including the proposed one. First, it can be seen from the table that under the same manufacturing process, although the work proposed by [7] had the highest detection accuracy, their system can only detect three types of ECG heartbeats: normal, atrial premature beat, and premature ventricular contraction; moreover, their chip area is also the largest of all works. In addition, the operating frequency of the circuit as proposed by [9] is the highest, but they could not achieve an adequately high accuracy (less than 95%). On the other hand, we may see from Table 5 that while the area of the chip as proposed in our study is much smaller than that as proposed in [7] (1.42 mm${}^{2}$ vs. 2.47 mm${}^{2}$), our chip is capable of identifying six types of ECG heartbeats with an adequately high accuracy of 96.83%, indicating that our work may achieve a better chip design than may other works. However, the weakness in our design is the proposed chip has higher power consumption than do the other ones. Note that the power dissipation can be substantially reduced simply by reducing the operating frequency [16].

### 3.3. Performance Evaluation for ECG Classification

To demonstrate the performance of the proposed CNN chip, twelve thousand ECG segments in total were utilized for training process to obtain the CNN weights, and three thousand ECG segments were used for testing process. The testing result in terms of detection accuracy was then used to verify the function of the circuit. As a result, the detection results produced by the proposed CNN chip showed that it can achieve 96.3% in overall detection accuracy for the task of ECG heartbeat classification. Table 6 provides the numbers of ECG segments used for training and testing processes, respectively, for each type of ECG heartbeat. In order the evaluate the performance of the proposed chip for ECG classification, a 6×6 confusion matrix, as shown in Table 7, was further adopted. In general, the confusion matrix compares the actual labeled values with those predicted by the proposed CNN model, and each row consists of the predicted values of the labeled heartbeat corresponding to that row. Table 7 provides the detection results obtained from the chip for all six labeled ECG heartbeats. One may see from Table 7 that the detection results obtained from the proposed chip for all the ECG events might achieve more than 90% in accuracy, indicating that the proposed CNN chip may be suitably applied for wearable healthcare monitoring devices.

## 4. Conclusions

In this study, a VLSI chip of the proposed CNN accelerator with the applications into the ECG heartbeat detection was implemented using a TSMC 0.18 μm COMS technology. The CNN circuit mainly consists of PE, ReLU, Softmax, MaxPooling, and Control Buffer modules, and all the modules can meet the requirement of repeated use so that the utilization may be maximized. As a result, it is revealed from the chip characteristics that the proposed CNN chip achieved a substantially small-area and high-speed design, in comparison to a number of previous works. Furthermore, the experimental results also demonstrated that the proposed chip can achieve 96.3% in overall detection accuracy for identifying six types of ECG heartbeats, and this would represent a significant benefit from this study. We may speculate that the proposed CNN chip may be applicable for the development and demand of wearable healthcare monitoring devices.

## Figures and Tables

**Table 1:** Listing of different types of ECG heart beats drawn from the MIT-BIH arrhythmia database to be detected.

Item	Diseases	Codename
1	Normal Beat	N
2	Left Bundle Branch Block Beat (LBBB)	L
3	Right Bundle Branch Block Beat (RBBB)	R
4	Premature Ventricular Contraction (PVC)	V
5	Atrial Premature Beat (APB)	A
6	Paced Beat	/

**Table 2:** Number of parameters in each layer.

Layer	Name	Number of Parameters
1	Convolution (3×1×7)	21
2	MaxPooling (3×1×2 )	0
3	Convolution (1×1×1 )	3
4	Convolution (3×1×7 )	21
5	MaxPooling (3×1×3 )	0
6	Flatten	0
7	Fully Connected (6×21 )	126
8	Fully Connected (21×6 )	126
	Total	297

**Table 3:** The designated values of the select input(s) of each multiplexer when operating at different layers.

Layer	Name	$S_{1}$	$S_{2}$	$S_{3}$	$S_{4}$	$S_{5}$	$S_{6}$
1	Convolution	0	1	×	×	×	×
2	MaxPooling	×	×	1	1	×	×
3	Convolution	1	0	×	0	00/01/10	00
4	Convolution	1	1	×	×	×	10
5	MaxPooling	×	×	0	1	×	×
6	Flatten	×	×	×	×	×	×
7	Fully Connected	1	1	×	0	×	11
8	Fully Connected	1	×	×	×	×	01

× means do not care.

**Table 4:** Chip characteristics of the proposed CNN accelerator.

Process Technology	TSMC CMOS 0.18-μm
Supply Voltage	1.8 V
Clock Frequency	60 MHz
Core Area	1.187×1.196 mm${}^{2}$
Power Consumption	4.4 mW @ 60 MHz
Latency	13.94μs

**Table 5:** Performance comparison of the proposed circuit and a number of existing works.

Method	[6]	[7]	[8]	[9]	Proposed
Database	MIT-PTB	MIT-BIH	MIT-BIH	MIT-BIH	MIT-BIH
# of Diseases	1	1	2	1	5
Accuracy	95.8%	97.25%	97.02%	94.94%	96.3%
Technology	90 nm	0.18 μm	0.18 μm	0.18 μm	0.18 μm
Voltage	0.5 V	1.2 V	1.0 V	1.8 V	1.8 V
Frequency	25 MHz	120 Hz	1 KHz	66.6 MHz	60 MHz
Area	4.90 mm${}^{2}$	2.47 mm${}^{2}$	N/A	0.73 mm${}^{2}$	1.42 mm${}^{2}$
Power	48.06 μW	5.97 μW	5.04 μW	3.1 mW	4.4 mW

**Table 6:** The numbers of ECG segments used for training and testing processes for each type of ECG heartbeats.

Item	Diseases	Codename	# of Train	# of Test	Total
1	Normal Beat	N	2000	500	2500
2	Left Bundle Branch Block Beat	L	2000	500	2500
3	Right Bundle Branch Block Beat	R	2000	500	2500
4	Premature Ventricular Contraction	V	2000	500	2500
5	Atrial Premature Beat	A	2000	500	2500
6	Paced Beat	/	2000	500	2500
			12,000	3000	15,000

**Table 7:** Detection results for all six labeled ECG heart beats.

	Predict	N	L	R	V	A	/
Labels							
N		496	0	0	1	2	1
L		0	492	0	7	1	0
R		1	0	488	7	4	0
V		2	1	2	486	7	2
A		4	0	16	16	464	0
/		1	0	0	3	1	495
Accuracy		99%	98%	98%	97%	93%	99%

## Data Availability

Not applicable.
